# Supplementary material for: The burden of T. solium cysticercosis and selected neuropsychiatric disorders in Mocuba district, Zambézia province, Mozambique
Source: PLoS Negl Trop Dis. 2022 Jul 14;16(7):e0010606. doi: 10.1371/journal.pntd.0010606 (PMC9321429; doi:10.1371/journal.pntd.0010606)
Supplement: Attachment — Submitted filename: Manuscript Cystinet PLOS NTD.docx [file pntd.0010606.s001.docx]

The burden of *T. solium* cysticercosis and selected neuropsychiatric disorders in Mocuba district, Zambézia province, Mozambique

Irene Langa^1,2^, Fernando Padama^3^, Noémia Nhancupe^1,2^, Alberto Pondja^4^, Delfina Hlashwayo^5^, Lidia Gouveia^6^, Dominik Stelzle^8,10^ , Clarissa Prazeres da Costa^7^, Veronika Schmidt^8,9^, Andrea S Winkler^8,9,^, Emília Virgínia Noormahomed^1,2,11^

^1^	Microbiology Department, Parasitology Laboratory, Faculty of Medicine, Eduardo Mondlane University, Maputo, Mozambique

^2^	Mozambique Institute of Health Education and Research, Maputo, Mozambique

^3^	Zambézia Operational Research Unit, Provincial Directorate of Health, Quelimane, Mozambique

^4^	Faculty of Veterinary, Eduardo Mondlane University, Maputo, Mozambique

^5^ Faculty of Sciences, Eduardo Mondlane University, Maputo, Mozambique

^6^ Mental health Department; National Public Health Directorate, Ministry of Health Maputo, Mozambique.

^7^	Institute for Medical Microbiology, Immunology and Hygiene, Center for Global Health, School of Medicine, Technical University of Munich, Germany

^8^	Department of Neurology, Center for Global Health, School of Medicine, Technical University of Munich, Germany

^9^	Centre for Global Health, Institute of Health and Society, University of Oslo, Oslo, Norway

^10^ Chair of Epidemiology, Department of Sport and Health Sciences, Technical University of Munich, Germany

^11^	Department of Medicine, Infectious Diseases Division, University of California, San Diego, USA

*	Corresponding author:

E-mail: delfinahlashwayo@gmail.com (DH)

**Abstract**

**Background:** *Taenia solium* (neuro-)cysticercosis is a neglected tropical disease often associated with epileptic seizures and reportedly with other neuropsychiatric disorders. This study aimed to evaluate the association of the disease with selected neuropsychiatric disorders (headache, epileptic seizures and psychosis) in Mocuba district, Mozambique.

**Methodology:** Between March and May 2018, we conducted a cross-sectional study among 1,086 participants aged 2 years or above in Mocuba district, Zambézia province, central Mozambique to assess the seroprevalence of human cysticercosis and risk factors for infection, as well as to explore its relation to selected neuropsychiatric disorders. Socio-demographic and clinical data were collected from each participant using a modified questionnaire designed by the Cysticercosis Working Group for Eastern and Southern Africa. Additionally, neuropsychiatric disorders, such as epileptic seizures, partial seizures, headache and psychosis were assessed using four vignettes. *T. solium* antigen and cysticercosis IgG in serum were detected using both *T. solium* antigen B158/B60 enzyme linked immunosorbent assay (ELISA) and LDBIO Cysticercosis Western Blot respectively.

**Principal Findings:** Overall, 112/1,086 participants (10.3%) were sero-positive for *T. solium* antigen or antibodies. Prevalence of antibodies (6.6%; n=72) was higher than of antigens (4.9%; n=54). In the questionnaires, 530 (49.5%) of participants reported chronic headache, 293 (27%) had generalized epileptic seizures (27%; n=293), 188 (18%) focal seizures and 183 (18.3%) psychosis. We found no statistically significant association between seropositivity for *T. solium* and the above-mentioned disorders. However, increasing age, being male and lack of knowledge regarding *T. solium* were associated with Ag-ELISA seropositivity.

**Conclusions:** Our study revealed that in Mocuba, *T. solium* cysticercosis is prevalent as neuropsychiatric disorders such as self-reported chronic headache, psychosis and epileptic seizures, however, independent of cysticercosis serology. Future studies are needed to confirm the burden of neuropsychiatric disorders and their possible etiology including neurocysticercosis with additional molecular and radiological diagnostic tools, as well as in-depth clinical examinations.

**Keywords:** *Taenia solium*; cysticercosis; neurocysticercosis; epilepsy; epileptic seizures; psychosis; headache

**Author summary:**

*Taenia solium* cysticercosis (TSC) is a neglected tropical disease caused by the larval stage of *T. solium*. The disease is a serious threat to public health, especially in low-income countries and is associated with poor pig husbandry practices, deficient hygiene and sanitation, close contact between humans and pigs, lack of slaughter facilities for pigs and inadequate meat inspection. When the larva of *T. solium* lodges in the human central nervous system, the disease is called neurocysticercosis (NCC). The most frequent neurological manifestations of NCC are epileptic seizures and epilepsy. NCC diagnostics remains a challenge in low-income countries and its relationship with neurological symptoms and signs was never studied in one of the districts with the biggest pig populations in Mozambique, Mocuba, located in Zambézia province. With this study we investigated for the first time the seroprevalence for TSC, risk factors for infection and the association with neuropsychiatric disorders. Seroprevalence of TSC was 10.3% and increasing age, being male and lack of knowledge regarding *T. solium* were associated with Ag-ELISA seropositivity. Thus, a point of care diagnostic tool should be developed in resource-poor settings such as Mocuba, and future studies are needed to analyze clinical and molecular aspects of the disease.

Introduction

*Taenia solium* cysticercosis (TSC) is a foodborne, zoonotic and neglected tropical disease (NTD), caused by the larval stage (cysticercus) of *T. solium.* The disease is emerging as a serious threat to public health, inflicting significant economic losses and disabilities in low-income countries of sub-Saharan Africa, Latin America and Asia where *T. solium* is endemic. It is also of increasing concern in non-endemic countries due to globalization and the immigration of tapeworm carriers [1-5].

Humans are the definitive host and thus carriers of the adult tapeworm which develops after ingestion of measly raw or undercooked pork. However, humans may also become intermediate hosts and develop TSC following direct or indirect ingestion of *T. solium* eggs which develop into cystic larvae in various parts of the body [1,5-8].

When the larva lodges in the central nervous system (CNS), the disease is called neurocysticercosis (NCC). The most frequent neurological manifestations include epileptic seizures and epilepsy. In endemic areas, up to 30% of people with epilepsy may suffer from NCC [1-5,9]. Other neurological signs and symptoms include severe headache, intracranial hypertension, dementia, blindness, or chronic meningitis. Also, different neuropsychiatric disorders have been associated with NCC although there is only limited data available [1,5,7,8,10].

The disease prevails due to the maintenance of *T. solium* life cycle, associated with poor pig husbandry practices, deficient hygiene and sanitation, close contact between humans and pigs, the intermediate host, lack of slaughter facilities for pigs and inadequate meat inspection [1,7,8,11,12]. However, the diagnosis of NCC remains a challenge because of the poor specificity of clinical and neuroimaging findings, the latter including magnetic resonance imaging (MRI) and computed tomography (CT scan), as well as suboptimal predictive values in immunodiagnostic tests, particularly in endemic settings [1,2].

The few available cross-sectional serological studies from Mozambique found that the seroprevalence of either antibodies or antigens against *T. solium* cysticercerci varied between 15% to 21% in apparently healthy children and adults, while in neuropsychiatric patients the seroprevalence of cysticercosis can be as high as 51% [13-15]. Further, the only published community-based study conducted in Angónia district, central western part of the country, found that amongst 151(8.8%) people confirmed to be living with epilepsy, 107 (70.9%) were seropositive to cysticercus antigens [14]. These data suggest that NCC might be potentially one of the main causes of epileptic seizures/epilepsy and other neuropsychiatric disorders in Mozambique.

Therefore, this study investigated the seroprevalence for *T. solium* cysticercosis and risk factors for infection as well as explored the association with neuropsychiatric disorders with an emphasis on epileptic seizures, headache and psychosis in both children and adults from Mocuba district, Zambézia province.

Methods

**Ethical Approval**

The present study was approved by the Mozambique National Bioethical Committee of Health ( 51/CNBS/2017) and by the administrative authorities from Zambézia province and community leaders. The study also received approval from the ethics committee of the Klinikum rechts der Isar, Technical University of Munich, Germany ( 537/18 S-KK).

Prior to the interviews and blood sampling, written and oral consent were obtained from the enrolled participants. Consent from children and illiterate participants were obtained in the presence of a guardian or witness, who signed on their behalf. Each participant was assigned a unique identification study number and all data and samples were handled confidentially using this study number from this point onward.

Participants who suffered from epileptic seizures/epilepsy or other neuropsychiatric disorders were referred to the local health centre for diagnosis confirmation and follow-up according to the Mozambique Ministry of Health guidelines.

**Study area and population**

The study was conducted in seven villages of Mocuba district (Fig 1) located in Zambézia province, central Mozambique (16°50' 13'' S, 36°59' 14'' E). The district has an area of 8,803 km^2^ and an altitude of 200–400 meters above sea level.

The total population is estimated at 422,681. There is an illiteracy rate of 62.5% with most of the population (82.6%) living in rural areas. Moreover, the disease profile of Mocuba is dominated by infectious diseases including many NTDs with higher prevalence estimates. As an example, the district has an HIV prevalence of 9% and 39% of children are infected by at least one intestinal parasite [16-20].

**Fig 1. Location of the study area.**

**Study design and sampling**

A community-based cross-sectional study was conducted between March and May 2018. The sampling frame was constructed using information of the National Institute of Statistics. The sampling procedure followed a two-stage household-based design, whereby Primary Sampling Units (PSUs) consisted of villages, and households were chosen during the second stage. Prior to selection of households, the village authorities were approached and the purpose of the study was explained. Once the permission was granted, a systematic random sampling with probability proportional to size (PPS) technique was used to select households. Two eligible participants from each household were recruited randomly into the study (Fig 2). Inclusion criteria were,living in the household and being at least 2 years old. People who had taken alcoholic beverages in the previous 24 hours were excluded.

**Questionnaire survey and vignettes**

A pre-designed questionnaire developed by the Cysticercosis Working Group for Eastern and Southern Africa (CWGESA) was used, with some modifications, that included four vignettes on neuropsychiatric disorders [21,22]. The questionnaire also covered information on socio-demographic and risk factors associated with cysticercosis such as age, gender, education, sources of drinking water, use of latrines, rearing pigs in the house and pig management practices, consumption of pork, awareness about TSC transmission, risk-related habits and practices. The four vignettes of the survey were chronic headache, epileptic seizures/epilepsy (generalised and focal), and psychosis.

Furthermore, direct observation to visualize the existence of latrines and usage and pig husbandry system in the household was made by the enumerators. The questionnaire designed in Portuguese language was used for the study, translated to the local language (Lomué) and back translated to Portuguese.

The survey was carried out by locally selected enumerators with knowledge of the local language, experience with community surveys, and basic training on TSC transmission, prevention and clinical signs/symptoms of selected neuropsychiatric disorders. The knowledge and training success of the enumerators was done via workshop sessions and pre-tested prior to commencement of the study.

**Fig 2. Data collection workflow of the study sample.**

**Vignette 1. Assessment of chronic headache**

To assess headaches, participants were asked if they had a history of daily headaches that occurred for at least 15 consecutive days and for longer than three months for no apparent reason or were associated with the most common causes of headaches such as malaria and hypertension.

**Vignette 2. Assessment of generalized tonic-clonic seizures**

To assess generalized tonic-clonic seizures (GTCS), we asked the participant if he/she had ever experienced a sudden loss of consciousness that caused him/her to fall to the ground or if he/she had noticed this. We also asked if the eyes were turned upwards, the body became rigid and/or involuntary violent movements of the arms and legs were observed. In addition, we asked if they were told or noticed that they were drooling and/or urinating and/or biting their tongue. We also asked if the person was asleep or slowed down or even confused after the actual epileptic seizure and could not remember what happened.

**Vignette 3. Assessment of focal seizures**

To assess focal seizures (FS), participants were asked whether they had ever noticed or been told about uncontrolled movements in body parts such as an arm or leg without loss of consciousness or before loss of consciousness. For the latter, we also asked about warning signs such as a strange smell (aura) before the epileptic seizure.

**Vignette 4. Assessment of psychosis disorders**

To assess psychotic behaviour and/or hallucinations, we asked the participant if he/she was informed of any changes in behaviour, such as becoming very unstable, appearing anxious and/or doing inappropriate things, such as undressing or harming others for no reason. They were also asked if they heard strange voices that no one else could hear, or if they saw things that were not real to others. They were also asked if they had the impression of being followed.

**Blood sampling**

A 5 ml sample of venous blood was obtained by venepuncture from each of the recruited individuals after consenting. The samples were immediately stored in cooling boxes at 4^°^C and transported to the Mocuba district Hospital Laboratory, where they were centrifuged for 5 min at 1500 r.p.m. to obtain serum. Serum was stored at -20 °C until shipment to the Parasitology Laboratory at Faculty of Medicine, Eduardo Mondlane University (UEM) in Maputo where samples were stored at -80 °C until further processing.

**Serological testing**

*T. solium* circulating antigens (Ag) were detected using the monoclonal antibody based B158C11A10 Enzyme Linked Immunosorbent Assay (Cysticercosis Ag-ELISA, ApDia®, Belgium). Ag index was calculated as the mean of optical density (OD) of each serum sample divided by the cut-off. In addition, IgG antibodies (Ab) against *T. solium* larva were determined via *Western Blot* (LDBIO Diagnostic®, Lyon, France). A sample was considered positive if at least 2 bands (6-8 kDa; 12 kDa; 23-26 kDa; 39 kDa; 50-55 kDa) were visible, according to the manufacturer’s instructions.

**Statistical analysis**

Data were captured in EpiData Entry Client version 4.3 and analysed using STATA version 13.0 (Stata Corp., College Station, TX). Descriptive analyses were first performed by calculating frequencies and percentages for categorical variables. Age was categorized into age groups (2–14, 15–24, 25–54 and >55 years) for the purpose of analysis. Bivariate analysis was performed to assess associations between a positive Ag-ELISA and/or *Western Blot* assay results, the socio-demographic and neuropsychiatric/neurological diseases, knowledge about *T. solium* as well as transmission risk-related variables and pig management variables using Chi-square test. Logistic regression models were performed to assess the association of seroprevalence of *T. solium* with the four vignettes. Models were run unadjusted and adjusted for age, gender, education and occupation. The significance level was set at p<0.05.

Results

A total of 1,086 participants from 565 households were included the study. The age of the participants ranged from 2 to 87 years, with a median age of 25 years. Among the recruited participants, 656 (60.4%) were predominantly farmers, 640 (58.9%) were female, and the majority 763 (70.3%) attended primary school. A total of 237 (21.8%) participants reported outdoor defecation, 806 (74.2%) reported eating pork, 155 (14.3%) were pig farmers and 105 (9.7%) have heard about the pork tapeworm (Table 1).

**Table 1. Socio-demographic characteristics of the study participants.**

The results of cysticercosis serological screening either for antigen or antibody detection or both from each village are shown in Fig 3. Overall, out of the 1,086 samples tested, 112 (10.3%) were positive in at least one serological test; 54 (4.9%) had circulating antigens and 72 (6.6%) had circulating antibodies to *T. solium* larva. Moreover, 14 (1.3%) were positive in both tests. A wide variation of seropositivity was observed across different villages ranging from 3.6% in Alto Benfica village to 17.7% in Mocuba Municipality.

In general, the seroprevalence of Ab tended to be higher (ranging from 3.6% in Alto Benfica village to 9.5% in Mocuba Municipality) than that of Ag (ranging from 0% in Munhiba to 8.2% in Mocuba Municipality). The details about serological assay results from each of the studied villages can be seen in Figure 3.

**Fig 3. Prevalence of human cysticercosis according to each study village in Mocuba district.**

The analysis of socio-demographic variables in relation to the cysticercosis serological assays are presented in Table 2. Seropositivity for antigen or antibody increased with age and was lowest for children under the age of 15 years (p=0.03). Seropositivity was higher for males than females (11.7% versus 9.4%, p=0.22) and increased with increasing level of education from 6.1% among people with no formal education to 15.7% among people with secondary or higher education (p=0.12), although there were no statistically significant differences.

**Table 2. Socio-demographic data and relation with serological assays to *T. solium* larva antigens and antibodies.**

The findings concerning the assessment of clinical signs and symptoms related to neuropsychiatric disorders in relation to socio-demographic factors are presented in Table 3. A total of 530 (49.5%) participants screened positive for vignette 1. More males screened positive than females (p=0.02). Education was not associated with screening positive but farmers more commonly screened positive than people with other occupations (p=0.05). Overall, 293 participants (27%) screened positive for vignette 2; positivity decreased with increasing age and was similar for males and females. Vignette 3 was positive in 188 (18%) of the participants and vignette 4 in 183 (18.3%). Vignette 4 was more commonly positive among participants older than 14 years and was more commonly positive among farmers. None of the vignettes were associated with seropositivity, neither in crude nor adjusted analyses (Table 4).

**Table 3. Assessment of clinical signs and symptoms related to neuropsychiatric disorders in relation to socio-demographic factors.**

**Table 4. Association between screening positive for vignettes and seropositivity.**

Discussion

To the best of our knowledge, this was the first pilot study performed in the country, particularly in Mocuba district with the aim to generate baseline information about seroprevalence and risk factors for human cysticercosis, and its possible relationship with some selected neuropsychiatric disorders. There were several important findings from our study.

**Serology**

our findings revealed that human cysticercosis was highly prevalent in the setting of our study although lower than previously reported in Angónia district and Beira city,Central Mozambique. These studies found in general higher prevalence of either antigens (15%) or antibodies (10%) to *T. solium* larva than the ones reported in this study [14,15], and were consistent with studies done in other settings such as Cameroon [23], Nigeria [24], Vietnam [25,26], Zambia [27] and Democratic Republic of Congo [5], which reported similar seroprevalence rates. Only 12.5% of the screened sera tested simultaneously positive to both serological assays. The differences in the prevalence of antigens (current infection) and antibodies (previous or current exposure to infection) may be influenced by the number, location of cysts and their larval stage. Furthermore, antibodies can persist longer, even after the clearance of the infection, while the presence of antigens is only detected when viable parasites are present [1-3,10,28,29]. Moreover, the western blot assay we used detected only three out of the seven glycoproteins described to be specific for cysticercosis [1,28,30]. So, it is possible that the prevalence of antibodies found in our study was underestimated.

In addition to that, the differences in study design, HIV serostatus characteristics of the study participants (immunocompetent or immunocompromised), serological assays used for each study, and parasite genetic diversity (which ultimately influenced the sensitivity and specificity of the serological assays) can explain the differences found within studies in the same region and in other settings [5,9,10,31]. Of note, that to date, we do not have systematic studies done in Mozambique accessing the existing genetic variants of *T. solium,* except for the one in which phylogenetic studies of cysts from Central Mozambique revealed the existence of the Latin variant [28]. As Mozambique has been exposed to migration from Europe and Asia, we cannot exclude the possibility that Mozambique has both variants circulating in different regions and further studies should be done in order to provide relevant information about this subject.

**Cysticercosis serology and neuropsychiatric disorders**

Secondly, our study also demonstrated that generalized epileptic seizures (27%), focal seizures (18%), headache (49.5%) and psychosis (18.3%) affected a substantial proportion of study participants irrespective of their cysticercosis serostatus . Our findings are consistent with data from 2018 hospital records , indicating that Mocuba district had the highest number of patients (7,416; 75.3%) in the entire province (9,844) with mental disorders. Out of these 7,416 mental health out-patients, 1,863 (25.1%) had epilepsy, 250 (3.4%) had mental retardation and 1,401 (18.9%) had psychosis [32].

In our study, epileptic seizures were significantly more prevalent in younger participants (2 – 14 years) and decreased with age (p<0.001). This finding is consistent with recent mental health data in Mozambique were it was found that among people living with epilepsy, 54% were children aged up to 15 years, though the underlying causes of these conditions are not well defined [20,33]. Similar studies in African countries also found that epileptic seizures and epilepsy were the main leading causes of mental health problems in out-patients’ consultations and that the majority of people affected are children and adolescents [14,16,33,34]. Although we did not find significant associations between serology and epileptic seizures, as with headache and psychosis, there was a tendency towards a higher percentage of people with epileptic seizures being positive for antibodies compared to antigens. Our findings therefore present a very high prevalence of epileptic seizures (both generalised and focal) that occur independently of seropositivity to cysticercosis and thus could be caused by other underlying diseases such as cerebral malaria, head trauma or perinatal hypoxia in children, to name a few.

When viewed in context of other similar studies, our findings here corroborate results from Bangoua,Cameroon [23], which did not find any statistically significant difference in the seropositivity to *T. solium* larva between people with epileptic seizures (1/61, 1.6%) and those without (5/323, 1.5%). Similarly, another study in the Democratic Republic of Congo [5] revealed that there was no difference in seropositivity between people with neurological disorders and those without. In addition, there was no association between cysticercosis and epilepsy in a study conducted in the Gambia [35]. Nevertheless in contrast, it is well documented in the literature from Latin America, Asia and Africa, including the study done in Angónia district, that seropositivity to circulating antigens or antibodies of cysticercosis were associated with epilepsy in community-based studies [14,36,37,38]. Furthermore, the odds ratio from a study in Nigeria revealed that people with epilepsy in the study area with a relatively high cysticercosis prevalence, were two times more likely to be seropositive than those without [24].

In our study, chronic headache was not significantly associated with circulating antigens which is consistent with other studies such as in residents of Anzoátegui state in Venezuela, Vietnam and Nabo Village in Tiandong Province, China, where it was found that frequent or chronic headache was independent of whether individuals were seropositive and seronegative to cysticercal antigens [39-41]. As with epileptic seizures, these findings may suggest another etiological cause of headache such as tension-type headache or reflect the pathogenesis of the disease as the neurological symptoms may only appear when viable cysts (parasite) starts to degenerate and eventually dies [1,2]. However, other studies done in Mozambique, Burkina Faso and Tanzania found that seropositivity to cysticercal antigens was associated with severe headache [14,15,38,42].

In addition to the high burden of epileptic seizures and chronic headache, we also found that psychotic manifestations were highly prevalent in the setting of our study (18.3%), confirming the anecdotal reports from the Mocuba district community leaders of having a high proportion of community residents with those conditions. As with the other two neuropsychiatric disorders, psychotic disorders do not seem to be related to cysticercosis seropositivity either. Psychotic disorders are understudied in Africa, as revealed by a recent scoping review [43], that found only nine studies in which the prevalence of psychotic disorders was analysed in Africa from 1984 to 2020. The available reports describe a prevalence ranging from 1% to 4.4% in rural regions. The only published population-based study done in 2003 in a rural (Cuamba) and urban (Maputo) areas of northern and southern Mozambique, respectively, found that seizure disorders (4.4% vs 1.6%), psychosis (4.4% vs 1.9%) and mental retardation (1.9% vs 1.3%) were higher in rural compared to urban areas [21] and well below the results of our study and recent hospital data from Zambézia Provincial Directorate [32]. It is difficult to explain this high burden. Apart from other CNS pathogens such as *Toxoplasma gondii*, *Toxocara spp*. and onchocerciasis (this last reported to exist in Zambézia province) [19,20,44,45], we think that the prolonged army conflict that affected this region for decades in some of the study villagers [46,47], combined with the stress it provokes in the affected population, might be playing a role as causative agent for this high prevalence of psychosis. Indeed, the effects of army conflict on mental health within the affected communities is well documented in the literature, as it causes disruption of socio-economic and political infrastructure including the appropriate delivery of health care services, which was the case in our study setting and other regions of Mozambique [46-49].

**Risk factors and multivariate logistic regression**

In our study, older age, being a male and those who have never heard about *T. solium* were more likely to be seropositive for the parasite. Regarding age, similarly to our results, studies from Tanzania [42], Ecuador [50], Zambia [27], Peru [51], Burkina Faso [52] and Vietnam [25], including a systematic review [53], revealed an association between increasing age and seropositivity to cysticercosis*,* except for a study conducted by Noormahomed et al. [15], in which age was not significantly associated with the seroprevalence of the helminths studied, including cysticercosis.

Furthermore, there was no statistically significant difference between gender and seropositivity in Ecuador [50] and Zambia [27]. In Tanzania [42], females were more likely to be seropositive, while in Burkina Faso and Vietnam there were males [25,52]. On the other hand, neither gender nor age presented a statistically significant association with seropositivity to cysticercosis in other studies [26,39,41,54].

Analysing the knowledge of *T. solium,* our results are in alignment with the findings in other countries such as Nigeria [24] where poor knowledge of cysticercosis and improper pork preparation were identified as main risk and behavioural factors contributing to the high prevalence.

Other potential risk factors such as consumption of pork [55], pig keeping, use of unsafe water, faecal disposal and types of latrine were not statistically significant in our study, similarly to the study done in Angónia district [14]. This was expected since the study participants share the same environmental conditions, and are equally exposed to the same risk factors and consequently equally exposed to the infection.

In some studies, food, water and hygiene related factors were significantly associated with cysticercosis seropositivity, including consumption of undercooked pork [41], and using the same water source [42]; although in other studies, factors such as consumption of raw meat and/or vegetables [26] and hygiene (presence of latrine) [51] were not associated with cysticercosis seropositivity.

**Strengths and limitations**

The most outstanding strength of this study is reflected in the fact that, to the best of our knowledge, this is the first assessment done in Mozambique aiming to define the seroprevalence of cysticercosis and its possible association with some selected neuropsychiatric disorders.

It is therefore the first pilot community-based study representative of the Mocuba district population including rural and urban settings and all age groups except those under 2 years old; it provides data about seroprevalence of cysticercosis and prevalence of some neuropsychiatric disorders for which such data has never been collected; it used a standardized questionnaire and vignettes to study the prevalence of neuropsychiatric disorders.

The limitations included: The assessment of neuropsychiatric disorders was based on a questionnaire and vignettes designed for this purpose and not followed by other sophisticated structured interviews, clinical examination as well as use of other means of diagnosis required to confirm the clinical diagnosis; patients with epileptic seizures either with positive or negative serology for cysticercosis were not subjected to CT scan or MRI in order to confirm or not the diagnosis of NCC; the serological assays used have their limitations in terms of sensitivity and specificity as well as on the discrimination of past or current infection; serological screening for other pathogens such as malaria, toxoplasmosis, and toxocariasis, onchocerciasis that may cause the selected neuropsychiatric disorders that we studied was not done, thus it is not possible to draw firm conclusions about the fraction of participants whose symptoms could have been due to cysticercosis/NCC or its sequelae.

Conclusions

In conclusion, our study found a high seroprevalence of *T. solium* antigens and antibodies, although the association with epileptic seizures, chronic headache and psychosis, the prevalence of which was also increased, could not be established. These factors should be further investigated to determine their role in the aetiology of neuropsychiatric disorders in cysticercosis endemic areas. In addition, other CNS pathogens should be studied to define their role in the burden of our targeted neuropsychiatric disorders as well as to help in the differential diagnosis of cysticercosis/NCC. This should be done in combination with studies of psychological, economic and societal impact of armed conflicts in the region and in sub-Saharan Africa as a whole.

Given the limited value of serology for diagnosis of cysticercosis and NCC and the scarcity and costliness of imaging techniques in endemic countries, research priorities should be given to the development of a point-of-care diagnostic tool and/or a biomarker. This should be done in combination with studies on parasite genetic diversity, eco-epidemiological and clinical features of cysticercosis/NCC to assess their geographical distribution and patterns to help in the surveillance, diagnosis and control of cysticercosis and NCC.

**Acknowledgements**

We are indebted to the Zambézia Provincial Governor, Dr Abdul Razak Noormahomed and his team and to the community leaders whose support and collaboration during the field work was critical for the engagement of the study participants in this research and to the Mental health Department; National Public Health Directorate, Ministry of Health Maputo, Mozambique for providing clinical assistance to the participants. We also thank the study participants who consented to participate in this study.

**References**

1. Carpio A, Fleury A, Romo ML, Abraham R. Neurocysticercosis: the good, the bad, and the missing. Expert Rev Neurother. 2018;18:289-301. doi:10.1080/14737175.2018.1451328.
2. Del Brutto OH. Neurocysticercosis in Western Europe: a re-emerging disease? Acta Neurol Belg. 2012;112:335-343. doi:10.1007/s13760-012-0068-3.
3. Garcia HH, Nash TE, Del Brutto OH. Clinical symptoms, diagnosis, and treatment of neurocysticercosis. Lancet Neurol. 2014;13:1202-1215. doi:10.1016/S1474-4422(14)70094-8.
4. Nhancupe N, Noormahomed EV, Afonso S, Svard S, Lindh J. Further evaluation of recombinant Tsol-p27 by enzyme-linked immunoelectrotransfer blot for the serodiagnosis of cysticercosis in pigs from Mozambique. Parasit Vectors. 2019;12:564. doi:10.1186/s13071-019-3816-x.
5. Mukendi D, Kalo JL, Lutumba P, Barbé B, Jacobs J, Yansouni CP, et al. High frequency of Taenia solium antigen positivity in patients admitted for neurological disorders in the Rural Hospital of Mosango, Democratic Republic of Congo. BMC Infect Dis. 2021;17:21(1):359. doi: 10.1186/s12879-021-06032-8.
6. Flisser A, Madrazo I, Plancarte A, Schantz P, Allan J, Craig P, et al. Neurological symptoms in occult neurocysticercosis after single taeniacidal dose of praziquantel. Lancet. 1993; 342:748. doi:10.1016/0140-6736(93)91743-6.
7. Winkler AS. Neurocysticercosis in sub-Saharan Africa: a review of prevalence, clinical characteristics, diagnosis, and management. Pathog Glob Health. 2012; 106:261-274. doi:10.1179/2047773212Y.0000000047.7.
8. Winkler AS, Willingham AL 3rd, Sikasunge CS, Schmutzhard E. Epilepsy and neurocysticercosis in sub-Saharan Africa. Wien Klin Wochenschr. 2009;121 Suppl 3:3-12. doi:10.1007/s00508-009-1242-3.
9. Ndimubanzi PC, Carabin H, Budke CM, Nguyen H, Qian YJ, Rainwater E, et al. A systematic review of the frequency of neurocyticercosis with a focus on people with epilepsy. PLoS Negl Trop Dis. 2010;4:e870. doi:10.1371/journal.pntd.0000870.
10. Garcia HH. Neurocysticercosis. Neurol Clin. 2018;36:851-864; doi:10.1016/j.ncl.2018.07.003.
11. Willingham AL 3rd,Engels D. Control of Taenia solium cysticercosis/taeniosis. Adv Parasitol. 2006;61:509-566. doi:10.1016/S0065-308X(05)61012-3.
12. Winkler AS. Epilepsy and neurocysticercosis in sub-Saharan Africa. In: Foyaca-Sibat H, editor. Novel aspects on cysticercosis and neurocysticercosis. InTech; 2013; p. 307-340.
13. Afonso SM, Vaz Y, Neves L, Pondja A, Dias G, Willingham AL, et al. Human and porcine Taenia solium infections in Mozambique: identifying research priorities. Anim Health Res Rev. 2011;12:123-129. doi:10.1017/S1466252311000077.
14. Assane YA, Trevisan C, Schutte CM, Noormahomed EV, Johansen MV, Magnussen P. Neurocysticercosis in a rural population with extensive pig production in Angonia district, Tete Province, Mozambique. Acta Trop. 2017;165:155-160. doi:10.1016/j.actatropica.2015.10.018.
15. Noormahomed EV, Nhacupe N, Mascaro-Lazcano C, Mauaie MN, Buene T, Funzamo CA, et al. A cross-sectional serological study of cysticercosis, schistosomiasis, toxocariasis and echinococcosis in HIV-1 infected people in Beira, Mozambique. PLoS Negl Trop Dis. 2014;8:e3121. doi:10.1371/journal.pntd.0003121.
16. Noormahomed EV, Mandane A, Cuambe A, Rodrigues MA, Noormahomed S, Carrilho C, et al. Design and Implementation of Postgraduate Programs in Health in a Resource-Limited Setting in Mozambique (The Lurio University). Adv Med Educ Pract. 2021;12:399-412. doi:10.2147/AMEP.S291364.
17. Muadica AS, Balasegaram S, Beebeejaun K, Köster PC, Bailo B, Hernández-de-Mingo M, et al. (2021). Risk associations for intestinal parasites in symptomatic and asymptomatic schoolchildren in central Mozambique. Clin Microb Infect. 2021;27(4):624–629.
18. Grau-Pujol B, Massangaie M, Cano J, Maroto C, Ndeve A, Saute F, et al. Frequency and distribution of neglected tropical diseases in Mozambique: a systematic review. Infect Dis Poverty. 2019;8:103. doi:10.1186/s40249-019-0613-x.
19. Noormahomed EV, Akrami K, Mascaro-Lazcano C. Onchocerciasis, an undiagnosed disease in Mozambique: identifying research opportunities. Parasit Vectors. 2016;9:180. doi:10.1186/s13071-016-1468-7.
20. Noormahomed EV, Mascaro-Lazcano C. Onchocerciasis in Mozambique: An unknown condition for health professionals. EC Microbiol; 2019.15;160-167.
21. Patel V, Simbine, AP, Soares, IC, Weiss, HA, Wheeler, E. Prevalence of severe mental and neurological disorders in Mozambique: a population-based survey. Lancet. 2007;370:1055-1060. doi: 10.1016/S0140-6736(07)61479-2.
22. Cysticercosis Working Group in Eastern and Southern Africa. Declaration on Taenia solium cysticercosis/taeniosis. Acta Tropica. 2003;87:1-2. doi: 10.1016/S0001-706X(03)00049-4.
23. Nkouawa A, Dschanou AR, Moyou-Somo R, Sako Y, Ito A. Seroprevalence and risk factors of human cysticercosis and taeniasis prevalence in a highly endemic area of epilepsy in Bangoua, west Cameroon. Acta tropica. 2017;165:116–120. doi:10.1016/j.actatropica.2015.12.019.
24. Edia-Asuke AU, Inabo HI, Mukaratirwa S, Umoh VJ, Whong CM, Asuke S, et al. Seroprevalence of human cysticercosis and its associated risk factors among humans in areas of Kaduna metropolis, Nigeria. J Infect Dev Ctries. 2015;9:799-805. doi:10.3855/jidc.5415.
25. Nguyen T, Cheong FW, Liew JW, Lau YL. Seroprevalence of fascioliasis, toxocariasis, strongyloidiasis and cysticercosis in blood samples diagnosed in Medic Medical Center Laboratory, Ho Chi Minh City, Vietnam in 2012. Parasit Vectors. 2016;9(1):486. doi: 10.1186/s13071-016-1780-2.
26. Binh VTL, Dung DT, Vinh HQ, Anke VH, Nicolas P, Pierre D, et al. Human Taeniasis and Cysticercosis and Related Factors in Phu Tho Province, Northern Vietnam. Korean J Parasitol 2021;59(4):369-376. doi: 10.3347/kjp.2021.59.4.369.
27. Mwape KE, Phiri IK, Praet N, Muma JB, Zulu G, Van den Bossche P, et al. Taenia solium Infections in a rural area of Eastern Zambia-a community based study. PLoS Negl Trop Dis. 2012;6(3):e1594. doi: 10.1371/journal.pntd.0001594.
28. Ito A, Yamasaki H, Nakao M, Sako Y, Okamoto M, Sato MO, et al. Multiple genotypes of Taenia solium--ramifications for diagnosis, treatment and control. Acta Trop. 2003;87:95-101. doi:10.1016/s0001-706x(03)00024-x.
29. Palafox-Fonseca H, Zuniga G, Bobes RJ, Govezensky T, Pinero D, Texco-Martinez L, et al. Genetic variation in the Cytb gene of human cerebral Taenia solium cysticerci recovered from clinically and radiologically heterogeneous patients with neurocysticercosis. Mem Inst Oswaldo Cruz. 2013;108:914-920. doi:10.1590/0074-0276130308.
30. Tsang VC, Brand JA, Boyer AE. An enzyme-linked immunoelectrotransfer blot assay and glycoprotein antigens for diagnosing human cysticercosis (Taenia solium). J Infect Dis. 1989;159: 50-59. doi:10.1093/infdis/159.1.50.
31. Rodriguez S, Wilkins P, Dorny P. Immunological and molecular diagnosis of cysticercosis. Pathog Glob Health. 2012,106:286-298. doi:10.1179/2047773212Y.0000000048.
32. Direcção Provincial de Saúde da Zambézia, Programa de Saúde Mental. 2019. Relatório annual de Saúde Mental.
33. Wagenaar BH, Cumbe V, Raunig-Berho M, Rao D, Kohrt BA, Stergachis A, et al. Outpatient mental health services in Mozambique: use and treatments. Psychiatr Serv. 2016; 67:588-590. doi:10.1176/appi.ps.201500508.
34. Dos Santos PF, Cumbe V, Gouveia ML, de Fouchier C, Teuwen D, Dua T. Implementation of mhGAP in Mozambique: integrating epilepsy care into the primary health care system. Int J Ment Health Syst. 2019;13:36. doi:10.1186/s13033-019-0296-5.
35. Secka A, Marcotty T, De Deken R, Van Marck E, Geerts S. Porcine cysticercosis and risk factors in the Gambia and Senegal. J Parasitol Res. 2010;823892. doi:10.1155/2010/823892.
36. Quet F, Guerchet M, Pion SD, Ngoungou EB, Nicoletti A, Preux PM. Meta-analysis of the association between cysticercosis and epilepsy in Africa. Epilepsia. 2010;51:830-837. doi:10.1111/j.1528-1167.2009.02401.x.
37. Debacq G, Moyano LM, Garcia HH, Boumediene F, Marin B, Ngoungou EB, et al. Systematic review and meta-analysis estimating association of cysticercosis and neurocysticercosis with epilepsy. PLoS Negl Trop Dis. 2017;11:e0005153. doi:10.1371/journal.pntd.0005153.
38. Sahlu I, Carabin H, Ganaba R, Preux PM, Cisse AK, Tarnagda Z, et al. Estimating the association between being seropositive for cysticercosis and the prevalence of epilepsy and severe chronic headaches in 60 villages of rural Burkina Faso. PLoS Negl Trop Dis. 2019;13:e0007101. doi:10.1371/journal.pntd.0007101.
39. Chung JY, Eom KS, Yang Y, Li X, Feng Z, Rim HJ, et al. A seroepidemiological survey of Taenia solium cysticercosis in Nabo, Guangxi Zhuang Autonomous Region, China. Korean J Parasitol. 2005;43(4):135-9. doi: 10.3347/kjp.2005.43.4.135.
40. Trung DD, Praet N, Cam TD, Lam BV, Manh HN, Gabriël S, et al. Assessing the burden of human cysticercosis in Vietnam. Trop Med Int Health. 2013;18(3):352-6. doi: 10.1111/tmi.12043.
41. Toquero M, Morocoima A, Ferrer E. [Seroprevalence and risk factors of cysticercosis in two rural communities in Anzoátegui state, Venezuela]. Biomedica. 2017;1,37(0):66-74. Doi. 10.7705/biomedica.v37i2.2841.
42. Mwanjali G, Kihamia C, Kakoko DV, Lekule F, Ngowi H, Johansen MV, et al. Prevalence and risk factors associated with human Taenia solium infections in Mbozi District, Mbeya Region, Tanzania. PLoS Negl Trop Dis. 2013;7(3):e2102. doi: 10.1371/journal.pntd.0002102.
43. Greene MC, Yangchen T, Lehner T, Sullivan PF, Pato CN, McIntosh A, et al. The epidemiology of psychiatric disorders in Africa: a scoping review. Lancet Psychiatry. 2021;8(8):717-731. doi: 10.1016/S2215-0366(21)00009-2.
44. Forrester JV, McMenamin PG, Dando SJ. CNS infection and immune privilege. Nat Rev Neurosci. 2018;19(11):655-671. doi: 10.1038/s41583-018-0070-8.
45. Manuel L, Santos-Gomes G, Noormahomed EV. Human toxoplasmosis in Mozambique: gaps in knowledge and research opportunities. Parasit Vectors. 2020;13:571. doi:10.1186/s13071-020-04441-3.
46. Noormahomed EV, Mocumbi AO, Ismail M, Carrilho C, Patel S, Nguenha A, et al. The medical education partnership initiative effect on increasing health professions education and research capacity in Mozambique. Ann Glob Health. 2018;84:47-57. doi:10.29024/aogh.14.
47. Nachega JB, Grimwood A, Mahomed H, Fatti G, Preiser W, Kallay O, et al. From Easing Lockdowns to Scaling Up Community-based Coronavirus Disease 2019 Screening, Testing, and Contact Tracing in Africa-Shared Approaches, Innovations, and Challenges to Minimize Morbidity and Mortality. Clin Infect Dis. 2021;72:327-331. doi:10.1093/cid/ciaa695.
48. Cliff J, Noormahomed AR. Health as a target: South Africa's destabilization of Mozambique. Soc Sci Med. 1988;27:717-722. doi:10.1016/0277-9536(87)90331-5.
49. Morina N, Akhtar A, Barth J, Schnyder, U. Psychiatric Disorders in Refugees and Internally Displaced Persons After Forced Displacement: A Systematic Review. Front Psychiatry. 2018;9:433. doi:10.3389/fpsyt.2018.00433.
50. Praet N, Speybroeck N, Rodriguez-Hidalgo R, Benitez-Ortiz W, Berkvens D, Brandt J, et al. Age-related infection and transmission patterns of human cysticercosis. Int J Parasitol. 2010;40(1):85-90. doi: 10.1016/j.ijpara.2009.07.007.
51. Moyano LM, O'Neal SE, Ayvar V, Gonzalvez G, Gamboa R, Vilchez P, et al. High Prevalence of Asymptomatic Neurocysticercosis in an Endemic Rural Community in Peru. PLoS Negl Trop Dis. 2016;10(12):e0005130. doi: 10.1371/journal.pntd.0005130.
52. Carabin H, Millogo A, Cisse A, Gabriel S, Sahlu I, Dorny P, et al. Prevalence of and Factors Associated with Human Cysticercosis in 60 Villages in Three Provinces of Burkina Faso. PLoS Negl Trop Dis. 2015;9:e0004248. doi:10.1371/journal.pntd.0004248.
53. Coral-Almeida M, Gabriël S, Abatih EN, Praet N, Benitez W, Dorny P. Taenia solium Human Cysticercosis: A Systematic Review of Sero-epidemiological Data from Endemic Zones around the World. PLoS Negl Trop Dis. 2015;9(7): e0003919. doi:10.1371/journal.pntd.0003919.
54. Goodman KA, Ballagh SA, Carpio A. Case-control study of seropositivity for cysticercosis in Cuenca, Ecuador. Am J Trop Med Hyg. 1999;60(1):70-4. doi: 10.4269/ajtmh.1999.60.70.
55. Rebecca WP, Eugene II, Joshua K. Seroprevalence of antibodies (IgG) to Taenia solium among pig rearers and associated risk factors in Jos metropolis, Nigeria. J Infect Dev Ctries. 2013; 7:67-72. doi: 10.3855/jidc.2309.

**Funding**

This research work was funded by the German Federal Ministry of Education and Research (BMBF) under CYSTINET-Africa project number 01KA1618, grant number 81203599. Furthermore, the manuscript writing and publication was supported by the Fogarty International Center, Office of the Director, Eunice Kennedy Shriver National Institute of Child Health & Human Development and National Institute of Neurological Disorders and Stroke of the National Institutes of Health under Award Number D43TW010135. The content is solely the responsibility of the authors and does not necessarily represent the official views of the Fogarty International Center or the National Institutes of Health.

**Authors' contributions**

IL, FP, NN and EVN, delivered the interventions, supervised and monitored the household survey interviewers, performed the laboratory work contributed to data management, read, and approved the final manuscript. AP, CPC, VS, ASW and EVN conceived and designed the study, conducted the study, interpreted the data and reviewed the manuscript. In addition, AP, DS, oversaw data management, did the statistical analysis and interpretation, and also contributed to the writing of the manuscript. LG, DH contributed to the manuscript writing. All authors read and approved the final version of the manuscript.

**Tables**

**Table 1.** **Socio-demographic characteristics of the study participants.**

| **Variables** | **Study participants** | |
| --- | --- | --- |
|  | **n** | **(%)** |
| **Age group (years)** |  |  |
| 2 – 14 | 277 | 25.5 |
| 15 – 24 | 251 | 23.1 |
| 25 – 54 | 457 | 42.1 |
| ≥ 55 | 101 | 9.3 |
| **Gender** |  |  |
| Male | 446 | 41.1 |
| Female | 640 | 58.9 |
| **Level of education** |  |  |
| Illiterate | 197 | 18.1 |
| Primary | 763 | 70.3 |
| Secondary | 121 | 11.1 |
| Other | 5 | 0.5 |
| **Occupation** |  |  |
| Farmer | 656 | 60.4 |
| Trading | 28 | 2.6 |
| Student | 245 | 22.6 |
| State employee | 17 | 1.6 |
| Other | 140 | 12.9 |
| **Water source** |  |  |
| River | 82 | 7.6 |
| Well | 585 | 53.9 |
| Borehole | 405 | 37.3 |
| Tap | 14 | 1.2 |
| **Faecal disposal** |  |  |
| Latrine | 849 | 78.2 |
| Outdoor defecation | 237 | 21.8 |
| **Pork consumption** |  |  |
| Yes | 806 | 74.2 |
| No | 280 | 25.8 |
| **Pork preparation** |  |  |
| Boiled | 452 | 56.1 |
| Grilled | 345 | 42.8 |
| Other | 9 | 0.1 |
| **Pig keeping** |  |  |
| Yes | 155 | 14.3 |
| No | 931 | 85.7 |
| **Ever heard about tapeworm** |  |  |
| Yes | 105 | 9.7 |
| No | 981 | 90.3 |

**Table 2. Socio-demographic data and relation with serological assays to *T. solium* larva antigens and antibodies.**

| **Variables** | **Study**  **participants**  **n (%)** | **Ag-Elisa ^+^** | **Western blot ^+^** | **Ag-Elisa ^+^ and Western blot ^+^** | **Ag-Elisa ^+^ or**  **Western blot ^+^** | |
| --- | --- | --- | --- | --- | --- | --- |
|  |  | **n (%)** | **n (%)** | **n (%)** | **n (%)** | ***p*-value** |
| **Total** | 1086 | 54 (4.9) | 72 (6.6) | 14 (1.3) | 112 (10.3) |  |
| **Age group (years)** |  |  |  |  |  |  |
| 2 – 14 | 277 | 4 (1.4) | 13 (4.7) | 1 (0.4) | 16 (5.8) | 0.03 |
| 15 – 24 | 251 | 12 (4.8) | 17 (6.8) | 1 (0.4) | 28 (11.2) |  |
| 25 – 54 | 457 | 30 (6.6) | 37 (8.1) | 10 (2.2) | 57 (12.5) |  |
| ≥55 | 101 | 8 (7.9) | 5 (5.0) | 2 (2) | 11 (10.9) |  |
| **Gender** |  |  |  |  |  |  |
| Female | 640 | 26 (4.1) | 42 (6.6) | 6 (1.3) | 60 (9.4) | 0.22 |
| Male | 446 | 28 (6.3) | 30 (6.7) | 8 (1.3) | 52 (11.7) |  |
| **Level of education** |  |  |  |  |  |  |
| No formal education | 197 | 5 (2.5) | 7 (3.6) | 0 (0) | 12 (6.1) | 0.12 |
| Primary | 763 | 40 (5.2) | 55 (7.2) | 14 (1.8) | 81 (10.6) |  |
| Secondary or higher | 121 | 9 (7.4) | 10 (8.3) | 0 (0) | 19 (15.7) |  |
| Other | 5 | 0 (0.0) | 0 (0.0) | 0 (0) | 0 (0.0) |  |
| **Occupation** |  |  |  |  |  |  |
| Farmer | 656 | 38 (5.8) | 43 (6.6) | 11 (1.7) | 70 (10.7) | 0.63 |
| Other | 430 | 16 (3.7) | 29 (6.7) | 3 (0.7) | 42 (9.8) |  |
| **Water source** |  |  |  |  |  |  |
| River | 82 | 7 (8.5) | 5 (6.1) | 3 (3.7) | 9 (11.0) | 0.67 |
| Well | 585 | 28 (4.8) | 43 (7.4) | 6 (1) | 65 (11.1) |  |
| Borehole | 405 | 19 (4.7) | 22 (5.4) | 5 (1.2) | 36 (8.9) |  |
| Tap | 14 | 0 (0.0) | 2 (14.3) | 0 (0) | 2 (15.4) |  |
| **Faecal disposal** |  |  |  |  |  |  |
| Latrine | 849 | 47 (5.5) | 56 (6.6) | 13 (1.5) | 90 (10.6) | 0.56 |
| Outdoor defecation | 237 | 7 (3.0) | 16 (6.8) | 1 (0.4) | 22 (9.3) |  |
| **Pork consumption** |  |  |  |  |  |  |
| Yes | 806 | 43 (5.3) | 57 (7.1) | 3 (1.1) | 89 (11.0) | 0.18 |
| No | 280 | 11 (3.9) | 15 (5.4) | 11 (1.4) | 23 (8.2) |  |
| **Pork preparation** |  |  |  |  |  |  |
| Boiled | 452 | 21 (4.6) | 29 (6.4) | 7 (1.5) | 43 (9.5) | 0.13 |
| Grilled | 345 | 22 (6.4) | 28 (8.1) | 4 (1.2) | 46 (13.3) |  |
| Not specified | 9 | 0 (0.0) | 0 (0.0) | 0 (0) | 0 (0.0) |  |
| **Source of pork** |  |  |  |  |  |  |
| Informal market | 747 | 41 (5.5) | 52 (7.0) | 10 (1.3) | 83 (11.1) | 0.88 |
| Butchery | 2 | 0 (0.0) | 0 (0.0) | 0 (0) | 0 (0.0) |  |
| Other | 56 | 2 (3.6) | 5 (8.9) | 1 (1.8) | 6 (10.7) |  |
| **Pig keeping** |  |  |  |  |  |  |
| Yes | 155 | 10 (6.5) | 14 (9.3) | 3 (1.9) | 21 (13.5) | 0.15 |
| No | 931 | 44 (4.7) | 58 (6.2) | 11 (1.2) | 91 (9.8) |  |
| **Pig husbandry system** |  |  |  |  |  |  |
| Confinement | 52 | 6 (11.5) | 4 (7.7) | 1 (1.9) | 9 (17.3) | 0.33 |
| Free ranging | 103 | 4 (3.9) | 10 (9.7) | 2 (1.9) | 12 (11.7) |  |
| **Ever heard about the tapeworm** |  |  |  |  |  |  |
| Yes | 105 | 2 (1.9) | 6 (5.7) | 1 (1) | 7 (6.7) | 0.20 |
| No | 981 | 52 (5.3) | 66 (6.7) | 13 (1.3) | 105 (10.7) |  |

**Table 3.** **Assessment of clinical signs and symptoms related to neuropsychiatric disorders in relation to socio-demographic factors.**

| **Variables** | **Study**  **participants**  **n (%)** | **Vignette 1**  **(Chronic headache)** |  | **Vignette 2**  **Tonic clonic seizure)** | | **Vignette 3**  **(Focal seizure)** |  | **Vignette 4**  **(Psychotic disorder)** |  |
| --- | --- | --- | --- | --- | --- | --- | --- | --- | --- |
|  |  | **n (%)** | ***p*-value** | **n (%)** | ***p*-value** | **n (%)** | ***p*-value** | **n (%)** | ***p*-value** |
| **Total** | 1086 | 530/1070 (49.5) |  | 293/1086 (27) |  | 188/1042 (18) |  | 183/999 (18.3) |  |
| **Age group (years)** |  |  |  |  |  |  |  |  |  |
| 2 – 14 | 277 | 123/268 (45.9) | 0.47 | 89/277 (32.1) | <0.01 | 45/251 (17.9) | 0.55 | 23/219 (10.5) | <0.01 |
| 15 – 24 | 251 | 125/247 (50.6) |  | 73/251 (29.1) |  | 48/247 (19.4) |  | 49/242 (20.2) |  |
| 25 – 54 | 457 | 227/454 (50) |  | 116/457 (25.4) |  | 82/444 (18.5) |  | 92/441 (20.9) |  |
| ≥55 | 101 | 55/101 (54.5) |  | 15/101 (14.9) |  | 13/100 (13) |  | 19/97 (19.6) |  |
| **Gender** |  |  |  |  |  |  |  |  |  |
| Female | 446 | 197/438 (45) | 0.02 | 128/446 (28.7) | 0.32 | 72/424 (17) | 0.51 | 84/407 (20.6) | 0.14 |
| Male | 640 | 333/632 (52.7) |  | 165/640 (25.8) |  | 116/618 (18.8) |  | 99/592 (16.7) |  |
| **Level of education** |  |  |  |  |  |  |  |  |  |
| No formal education | 197 | 96/187 (51.3) | 0.74 | 60/197 (30.5) | 0.58 | 36/181 (19.9) | 0.90 | 23/157 (14.6) | 0.39 |
| Primary | 763 | 377/757 (49.8) |  | 199/763 (26.1) |  | 132/743 (17.8) |  | 136/718 (18.9) |  |
| Secondary or higher | 121 | 55/121 (45.5) |  | 32/121 (26.4) |  | 19/113 (16.8) |  | 24/119 (20.2) |  |
| Other | 5 | 2/5 (40) |  | 2/5 (40) |  | 1/5 (20) |  | 0/5 (0) |  |
| **Occupation** |  |  |  |  |  |  |  |  |  |
| Farmer | 656 | 338/650 (52) | 0.05 | 162/656 (24.7) | 0.04 | 113/646 (17.5) | 0.61 | 130/635 (20.5) | 0.03 |
| Other | 430 | 192/420 (45.7) |  | 131/430 (30.5) |  | 75/396 (18.9) |  | 53/364 (14.6) |  |
| **Water source** |  |  |  |  |  |  |  |  |  |
| River | 82 | 41/79 (51.9) | 0.80 | 23/82 (28) | 0.77 | 19/77 (24.7) | 0.24 | 12/73 (16.4) | 0.73 |
| Well | 585 | 292/582 (50.2) |  | 165/585 (28.2) |  | 102/560 (18.2) |  | 104/531 (19.6) |  |
| Borehole | 405 | 190/395 (48.1) |  | 101/405 (24.9) |  | 63/392 (16.1) |  | 66/382 (17.3) |  |
| Tap | 13 | 7/13 (53.8) |  | 4/13 (30.8) |  | 4/12 (33.3) |  | 1/12 (8.3) |  |
| Other | 1 | 0/1 (0) |  | 0/1 (0) |  | 0/1 (0) |  | 0/1 (0) |  |
| **Faecal disposal** |  |  |  |  |  |  |  |  |  |
| Latrine | 820 | 396/810 (48.9) | 0.57 | 220/820 (26.8) | 0.88 | 138/784 (17.6) | 0.62 | 132/765 (17.3) | 0.13 |
| Outdoor defecation | 237 | 121/231 (52.4) |  | 64/237 (27) |  | 46/230 (20) |  | 43/208 (20.7) |  |
| Other | 29 | 13/29 (44.8) |  | 9/29 (31) |  | 4/28 (14.3) |  | 8/26 (30.8) |  |
| **Pork consumption** |  |  |  |  |  |  |  |  |  |
| Yes | 280 | 127/276 (46) | 0.20 | 64/280 (22.9) | 0.08 | 40/269 (14.9) | 0.14 | 43/260 (16.5) | 0.44 |
| No | 806 | 403/794 (50.8) |  | 229/806 (28.4) |  | 148/773 (19.1) |  | 140/739 (18.9) |  |
| **Pork preparation** |  |  |  |  |  |  |  |  |  |
| Boiled | 452 | 192/446 (43) | <0.001 | 113/452 (25) | 0.04 | 72/431 (16.7) | 0.15 | 62/411 (15.1) | <0.01 |
| Grilled | 345 | 208/341 (61) |  | 112/345 (32.5) |  | 74/333 (22.2) |  | 77/320 (24.1) |  |
| Not specified | 9 | 3/7 (42.9) |  | 4/9 (44.4) |  | 2/9 (22.2) |  | 1/8 (12.5) |  |
| **Source of pork** |  |  |  |  |  |  |  |  |  |
| Informal market | 747 | 372/736 (50.5) | 0.36 | 211/747 (28.2) | 0.08 | 136/719 (18.9) | 0.49 | 128/688 (18.6) | 0.44 |
| Butchery | 2 | 2/2 (100) |  | 2/2 (100) |  | 1/2 (50) |  | 0/2 (0) |  |
| Other | 56 | 29/55 (52.7) |  | 16/56 (28.6) |  | 11/51 (21.6) |  | 12/48 (25) |  |
| **Pig keeping** |  |  |  |  |  |  |  |  |  |
| Yes | 155 | 66/153 (43.1) | 0.11 | 44/155 (28.4) | 0.74 | 27/146 (18.5) | 0.97 | 31/146 (21.2) | 0.39 |
| No | 931 | 464/917 (50.6) |  | 249/931 (26.7) |  | 161/896 (18) |  | 152/853 (17.8) |  |
| **Pig husbandry system** |  |  |  |  |  |  |  |  |  |
| Confinement | 52 | 21/52 (40.4) | 0.75 | 15/52 (28.8) | 1 | 9/49 (18.4) | 1 | 9/50 (18) | 0.63 |
| Free ranging | 103 | 45/101 (44.6) |  | 29/103 (28.2) |  | 18/97 (18.6) |  | 22/96 (22.9) |  |
| **Ever heard about the tapeworm** |  |  |  |  |  |  |  |  |  |
| Yes | 105 | 47/103 (45.6) | 0.47 | 34/105 (32.4) | 0.23 | 26/101 (25.7) | 0.05 | 20/102 (19.6) | 0.83 |
| No | 981 | 483/967 (49.9) |  | 259/981 (26.4) |  | 162/941 (17.2) |  | 163/897 (18.2) |  |

**Table 4. Association between screening positive for vignettes and serology positivity.**

| **Variables** | **Study**  **participants**  **n (%)** | **Ag-ELISA^+^ or**  **Western blot^+^** |  |  |
| --- | --- | --- | --- | --- |
|  |  | **n (%)** | ***Unadjusted Odds Ratio (95%CI)*** | ***Adjusted Odds Ratio (95%CI)⸸*** |
| **Total** | 1086 | 112 (10.3) |  |  |
| **Vignette 1** |  |  |  |  |
| No | 446 | 69 (12.8) | Reference | Reference |
| Yes | 640 | 43 (8.1) | 0.6 (0.4-0.9) | 0.61 (0.41-0.91) |
| **Vignette 2** |  |  |  |  |
| No | 656 | 85 (10.7) | Reference | Reference |
| Yes | 430 | 27 (9.2) | 0.85 (0.53-1.32) | 0.88 (0.55-1.38) |
| **Vignette 3** |  |  |  |  |
| No | 820 | 89 (10.4) | Reference | Reference |
| Yes | 29 | 20 (10.6) | 1.02 (0.6-1.68) | 1.04 (0.6-1.71) |
| **Vignette 4** |  |  |  |  |
| No | 280 | 87 (10.7) | Reference | Reference |
| Yes | 806 | 19 (10.4) | 0.97 (0.56-1.61) | 0.88 (0.5-1.46) |

⸸ Models were adjusted for age, gender, education and occupation
