# Supplementary material for: The burden of T. solium cysticercosis and selected neuropsychiatric disorders in Mocuba district, Zambézia province, Mozambique
Source: PLoS Negl Trop Dis. 2022 Jul 14;16(7):e0010606. doi: 10.1371/journal.pntd.0010606 (PMC9321429; doi:10.1371/journal.pntd.0010606)
Supplement: Attachment — Submitted filename: Response to reviewers PLOS.NTD REVIEW 1 26052022.docx [file pntd.0010606.s002.docx]

Dr. Ricardo Fujiwara

Deputy Editor

PLOS Neglected Tropical Diseases

PNTD-D-21-01739

Dear Dr. Ricardo Fujiwara,

We would like to thank for your favourable preliminary appraisal on Manuscript PNTD-D-21-01739, and for making available to us the reviewers' comments, which we have considered carefully. We thank the editors and the reviewers for a thorough reading and constructive criticism of our manuscript and for the opportunity to revise and resubmit. We are pleased to submit the revised and improved version of the manuscript for your consideration. We addressed point-by-point all the comments and issues raised by the reviewer that contributed for the improvement of the revised version of the manuscript. We have applied a tabular format, with the reviewer comments in the left column and our response on the right.

The authors thank the editors and reviewers and all concur for the resubmission of this versions; and we hope that now the manuscript is acceptable for publication. We appreciate your time and look forward to your response.

Sincerely,

Delfina Fernandes Hlashwayo

Corresponding author

Eduardo Mondlane University, Faculty of Sciences, Avenida Julius Nyerere, nr 3453, Maputo Mozambique. Tel: (+258) 21493377; email: [delfinahlashwayo@gmail.com](mailto:delfinahlashwayo@gmail.com)

May 13, 2022

| **No.** | **METHODS** | **RESPONSE** |
| --- | --- | --- |
|  | **Reviewer #1:** |  |
| 1. | - Line 49-50 in the abstract, should be corrected. | We thank the reviewer for the comment. We have corrected accordingly (Track changes: Abstract, line 50). |
| 2. | - Line 177 - I would ask the authors to specify the age of those participants considered children and, thus, needed the consent of a guardian. They should also specify if assent was required. | We considered as children all participants aged below 12 years. For participants aged between 12 and 17 years an assent was obtained in addition to their parent’s consent, according to the national bioethics committee guidelines. We have added this explanation in the manuscript (Track changes: Methodology, lines 192-194). |
| 3. | - The authors do not exclude those participants that recently moved into the area. They could have been infected in their previous residence. | We thank the reviewer for the comment. We consider this as methodological limitation in our study. |
| 4. | - Vignette 1-4: the authors should provide references. | We thank the reviewer for the comment. The vignettes were defined according to common specialist and textbook knowledge by the neurologist on the team (ASW) (Track changes: lines 233-236). |
| 5. | - Line 275-276: the authors do not adjust for having porks at home or in the neighbourhood. They should at least discuss how this could have affected in their results. They did not adjust for religion, what could have affected pork consumption. | Thank you for this comment. We have taken your comment and included pig keeping in the adjusted logistic regression models which yielded that pig keeping did not influence positivity in any of the four vignettes. Unfortunately, we did not have information available on religion. We agree this may have an effect on pork consumption which then again may have an effect on seroprevalence of *T. solium* or the vignettes. However, alike pig keeping, we do not think this variable would have influenced the results considerably. |
|  | **Reviewer #2:** |  |
| 1. | The objectives of the study were clear enough and had a clear testable hypothesis. The study design generally addressed the outlined objectives, what may have been considered an experimental lapse was explained in the limitations section. | We thank the reviewer for the comment. |
| 2. | The sample size was representative enough to cover the hypothesis being tested. Appropriate statistical tools were used, however, the statistical analysis done for table 4 is incomplete. I have recommended that a bivariate analysis be first of all done between the serostatus of cysticercosis and the vignettes described in the study and the results presented clearly, before going ahead with the binary regression analysis. | We thank the reviewer for this comment. We have now also included in Table 4 the p-values derived from the logistic regression models. These p-values differ only slightly from those derived from Chi square tests with/without Yates’ continuity correction which is why we did not additionally include p-values from Chi-square tests.  For example: p-value of the unadjusted logistic regression model with serology as outcome and vignette 1 as independent variable: 0.013; Chi-square test (serology and vignette 1) without Yates’ correction: 0.013; with Yates’ correction: 0.018. |
| 3. | Ethical permission/clearance was adequately sought and obtained. | We thank the reviewer for the comment. |

|  | **RESULTS** | **RESPONSE** |
| --- | --- | --- |
|  | **Reviewer #1:** |  |
| 1. | - Figure 2: English language should be reviewed. | We thank the reviewer for the comment. We revised the English language in Figure 2. |
| 2. | - Lines 293-295: Unclear if they used a statistical test to verify it. If it is not used, I recommend to use it. | Thank you for this comment. We have now tested the difference in antibody and antigen prevalence and the antibody prevalence is significantly higher than the antigen prevalence (p<0.005). Also, the seroprevalence differs significantly between districts (p=0.007). For both scenarios, Chi-Square tests were used. We have added this in the manuscript in lines 326-331 (track-changes). |
| 3. | - Line 300-303: This statement is not correct. | We thank the reviewer for the comment. We revised the statement (Track-changes: Results, lines 337-338). |
|  | **Reviewer #2:** |  |
| 1. | The findings of the study and the analysis plan do corroborate/match one another. Generally, the results were clear enough to comprehend, however, some of the data presented need to be reconciled. The statistical analysis for Table 4 has to be revisited. | We thank the reviewer for this comment. As mentioned above, we have now also included in Table 4 the p-values derived from the logistic regression models. |
|  | **CONCLUSIONS** | **RESPONSE** |
|  | **Reviewer #1:** |  |
| 1. | Results should be interpreted with care. | We thank the reviewer for the comment. We have improved our results section according to the reviewers comments in the word document. |
| 2. | - Lines 321-323 should verify is this statement is true. | We thank the reviewer for the comment. It is the first study in fact with the aim to generate baseline information about seroprevalence and risk factors for human cysticercosis and its possible relationship with some selected neuropsychiatric disorders. |
|  | **Reviewer #2:** |  |
| 1. | The conclusion needs to be rewritten to clearly convey the important information in the study. The major limitations of the study were mentioned by the authors. However, the authors did not discuss the public health relevance of the study. | We thank the reviewer for the comment. We have revised accordingly (Track-changes: Conclusions and recommendations, lines 545-552). |
|  | **EDITORIAL AND DATA PRESENTATION MODIFICATIONS?** | **RESPONSE** |
| 1. | Reviewer #1: English language | We thank the reviewer for the comment. We revised the English language in the manuscript. |
| 2. | Reviewer #2: I recommend that the manuscript be accepted subject to implementing the minor revisions recommended | We thank the reviewer for the comment. |
|  | **SUMMARY AND GENERAL COMMENTS** | **RESPONSE** |
|  | Reviewer #1: |  |
| 1. | This is very relevant data for Mozambique, since there is not much data on T. solium presence in this country. However, I would like to see some clarifying edits and corrections. | We thank the reviewer for the comment. We have revised and improved the manuscript according to the reviewers comments. |

|  | Reviewer #2: |  |
| --- | --- | --- |
| 1. | This is a relevant baseline study which has generated important information on human cysticercosis in some parts of Mozambique. The information could be used to inform policy and practice and also serve as a reference for prospective studies. | We thank the reviewer for the comment. |
| 2. | There was a flaw with some aspect of the experimental design that had to do with diagnosis of neuropsychotic disorders described in the studies, however, being a baseline community study, this could be overlooked, moreover, the authors actually mentioned this as a major limitation to their study. | We thank the reviewer for the comment. |
